# Supplementary material for: The effects of prolonged sitting, prolonged standing, and activity breaks on vascular function, and postprandial glucose and insulin responses: A randomised crossover trial
Source: PLoS One. 2021 Jan 4;16(1):e0244841. doi: 10.1371/journal.pone.0244841 (PMC7781669; doi:10.1371/journal.pone.0244841)
Supplement: S2 File — (DOCX) [file pone.0244841.s003.docx]

**Sitting, standing or breaking: Which is better for cardio-metabolic health?**

**A randomised cross-over study**

**Funding Agencies:** University of Otago Research Grant

**Principal Investigator:** Dr Meredith Peddie^1^

**Phone:** 03 479 8157

**Email:** [meredith.peddie@otago.ac.nz](mailto:meredith.peddie@otago.ac.nz)

**Co-investigators:** Dr Kate Thomas^2^

Associate Professor Nancy Rehrer^3^

Professor Jim Cotter^3^

^1^Department of Human Nutrition

^2^Surgical Sciences, Dunedin School of Medicine

^3^School of Physical Education, Sport ad Exercise Science

University of Otago

PO Pox 56

Dunedin

New Zealand

1. **Project Summary**

This study will provide important evidence around how reducing sedentary behaviour by standing, or performing short bouts of activity regularly may improve postprandial metabolism and endothelial function, both of which are important for cardio-metabolic health. We will conduct a randomised cross-over study involving 18 healthy, normal weight participants. The study will include three experimental intervention sessions: prolonged sitting; prolonged standing; and regular activity breaks, each separated by a minimum six-day washout period. Flow-mediated dilation will be measured at baseline, and at the conclusion of each intervention, while popliteal artery haemodynamics will be measured hourly to assess changes in blood flow and shear stress. Area under the curve will be calculated for plasma glucose and insulin. Mixed model regression will be used to examine differences in the effect of the three different interventions on markers of endothelial function and postprandial metabolism**.**

**2. Background**

Sedentary behaviour – activities performed in a seated or reclining position that involve very low energy expenditure (1) – predominate the waking hours of many adults. Recently we measured, using two different validated accelerometers, the sedentary behaviour patterns of 79 University of Otago employees and found, on average, they spend 75% of their workday sitting down (2). An increasing number of large-scale prospective studies have shown that sedentary time is associated with increased incidence of diabetes and cardiovascular disease and overall mortality (3, 4). Interestingly, the results of a recent meta-analysis indicate that only when 60-70 min of physical activity is performed on a daily basis are the negative consequences of sedentary behaviour overcome (5). This is a level of physical activity far above that of the University employees surveyed, despite many of them meeting currently physical activity guidelines (2).

Most intervention studies designed to test the causality of the association between sedentary behaviour and health outcomes have focused on the *acute* effects of regularly interrupting periods of prolonged sitting with short bouts of activity on postprandial metabolism. Indeed, our research group was among the first to provide experimental evidence indicating that breaking up periods of prolonged sitting with regular short bouts of activity markedly attenuates the rises in postprandial glucose and insulin concentrations observed with long periods of uninterrupted sitting, and that this attenuation is greater than that seen with a single bout of 30 min of continuous activity (6). A growing number of studies have now been conducted in this area, confirming that ~1.5 min – 5 min of light or moderate activity performed every 20 – 30 min can improve postprandial glucose metabolism when compared to 2 – 9 h of uninterrupted sitting in participants ranging from healthy inactive young adults to those who have obesity and type 2 diabetes (6-9). An alternative method for reducing prolonged sitting is to encourage standing. However, the evidence suggesting that standing desks are the best method for combating the perils of too much sitting lags markedly behind the recent explosion in sales of standing workstations (10). The results of a small number of acute studies indicate that short bouts of standing (11) or even alternating 30 min of standing with 30 min of sitting (12) are not as effective at reducing postprandial glycaemia as regular *activity* breaks, particularly in healthy, normal weight populations (11). Very little is known about the effects of more prolonged bouts of standing, despite recommendations being made to encourage individuals to stand for at least half of their work day (13).

The results of two recent studies performed using participants who have type 2 diabetes indicate that performing regular activity breaks over a seven-hour period during the day results in sustained improvements in nocturnal glycaemic control after the participant has left the laboratory (14, 15). However, it is not clear whether these effects would be observed in healthy people, or how long the improvements in glycaemic control are maintained. In addition, we have very little information about whether after a sustained period of performing regular activity breaks, or prolonged standing, individuals are likely to alter their activity levels to compensate for these different behaviours.

Endothelial function is an important and independent risk factor for cardiovascular disease and mortality (16). Change in localised haemodynamic forces (the forces associated with changing blood flow) play an important role in the development of endothelial dysfunction and atherosclerosis (17). Prolonged sitting reduces blood flow to the lower limbs, lowering shear stress (the force applied to the endothelial surface in the direction of the flowing blood), and resulting in transient endothelial dysfunction (18). Additionally, a greater volume of sedentary behaviour has also been associated with more permanent changes in endothelial function over time (19). It is likely that reductions in blood flow and endothelial function also contribute to the elevated postprandial glycaemia and lipidaemia observed with prolonged sitting given that muscle provides the largest source for glucose disposal, and that muscle prefusion facilitates glucose uptake. Reduced blood flow to the large muscles of the leg thereby reduces the major role those muscles can play in the regulation of glucose and lipid metabolism. To date, the results of two studies have indicated that both regular activity breaks (20) and standing (21) may protect against the negative impact sitting has on endothelial function. However, in both these studies the intervention period lasted only 3 hours, a duration of sitting much less than that accumulated during a usual work day (2). Additionally, there is some contrasting evidence to suggest that continuous standing may contribute to atherosclerosis progression in carotid arteries (22) – a result that would not be expected if standing was indeed having positive effects on blood flow and shear stress.

**3. Aim of Study**

To compare the effects of prolonged sitting, prolonged standing and sitting interrupted with regular short bouts of activity on postprandial glucose metabolism and endothelial function (via flow-mediated dilation).

**4. Objectives**

To compare sitting, standing and performing regular activity breaks for 6.5 h on their respective effects on

- flow mediated dilation and haemodynamics of the superficial popliteal artery;
- glucose and insulin AUC and iAUC; and
- the ensuing 48-h glucose profile and free-living activity patterns.

**5. Hypothesis**

We hypothesise that regular activity breaks, but not prolonged standing, will improve both endothelial function and postprandial glucose metabolism when compared to prolonged sitting.

**6. Study Design**

This study will be a randomized, three-way cross over trial, in which each participant will complete three 6.5-h intervention sessions: 1) Prolonged Sitting; 2) Prolonged Standing; and 3) Regular Activity Breaks, in which sitting will interrupted every 30 min with a 2-min bout of activity. Each intervention session will be separated by at least 6 days.

**7. Study Setting/ Location**

This single site study will be conducted in the School of Physical Education, Sport and Exercise Science at the University of Otago’s Dunedin Campus.

**8. Study Population**

18 healthy, normal weight adults between 18 and 40 years of age.

**9. Eligibility Criteria**

Participants in this study are required to be predominantly sedentary during the day, therefore we will require participants to:

- Self-report spending greater than 5 h per day, on average, engaged in sedentary behaviour;
- Not be currently using a sit-to-stand workstation on a regular basis.

Other research indicates that the effects of regular activity breaks on postprandial metabolism differ in people of different weight and health status (23), therefore, participants will only be included in the study if they:

- Have no personal history of cardiovascular disease or diabetes;
- are a non-smoker;
- Have a BMI of less than 35 kg·m^2^.

Because we are measuring postprandial glucose metabolism and endothelial function, which can be effected by medications, and dietary supplements, participants will also only be included if they are:

- Not taking any medication, dietary supplements, vitamins or minerals other than oral or other hormonal contraceptive agents.

We will also ask participants not to take part if:

- They have been told by a doctor that they should avoid doing physical activity or standing for long periods (because they will be asked to do both of these things in the study);
- They have an intolerance or allergy to dairy or gluten as the test meal and snack will contain these foods.

Participants will be excluded from participating if their Physical Activity Readiness Questionnaire (PAR-Q) data indicate that participating in physical activity may not be appropriate, or if their mean blood pressure measurement taken in the screening visit is greater than 140/90 mm Hg.

**10. Study Outcomes**

Primary Outcome: The difference in the change in flow-mediated dilation taken at 0 and 6 h between the three different interventions

Secondary Outcomes

1. The difference in the change over time in superficial femoral artery hemodynamics between the three different interventions.
2. The difference in glucose AUC and iAUC between the interventions.
3. The difference in insulin AUC and iAUC between the interventions.
4. The difference in the continuous glucose profile in the 48 following the interventions
5. The difference in free living activity patterns in the 48 h following the interventions

**11. Study Procedures**

Recruitment of participants

Paper and electronic copies of the recruitment flyers advertising the study will be distributed around the university campus. Individuals interested in participating will be asked to indicate interest by emailing activitybreaks-study@otago.ac.nz. The postgraduate research students involved in the study, or the PI (MCP) will respond to the email providing a copy of the information sheet and asking them to confirm that they meet the eligibility criteria. Participants who do not meet the eligibility criteria will be informed of this and thanked for their interest. Participants who apparently meet the eligibility criteria, and are still interested in participating upon reading the information sheet will be scheduled to attend an initial consent and screening visit at the Department of Human Nutrition. Recruitment will continue concurrently with data collection until 18 participants have completed the study.

Randomisation

Participants will be block randomized (block size n=6) to complete the three interventions in 1 of 6 possible orders. The randomization sequence will be generated by MCP using STATA software and concealed electronically. The evening before each participant begins his or her first intervention session MCP will reveal and assign the next sequential randomization. Participants will not be notified of which intervention session they are completing until the arrive at the clinic on the morning of each intervention session. However, by a process of elimination they will know what their third and final intervention will be as soon as they begin their second intervention session.

Study procedures

***Screening visit***

Once written informed consent has been obtained from the participant they will complete a questionnaire that asks their:

- Demographic information
- Heath history and status
- Physical Activity Readiness (PAR-Q)
- Usual physical activity patterns
- If they have an allergies/intolerances/strong dislikes of the study food

The participant will then have their height, weight and blood pressure (in triplicate 1 min apart; after 15-min seated rest) measured.

***Standardization of Prior Physical Activity and Diet***

To minimise the influence of previous activity on outcome measures, participants will be required to avoid all intentional moderate-to-vigorous intensity physical activity and be largely sedentary for 24 h prior to each intervention. In the 24 h before their first testing session, participants will be required to complete a 24-h diet record, in which they will record all food and beverages including caffeinated beverages. They will also be fitted with an accelerometer and interstitial glucose monitor to be worn for the following four days (from the day before the intervention through until 48 h post intervention). The diet record will be returned to participants 24 h prior to each subsequent intervention session, when they will also be refitted with an accelerometer. They will then be asked to replicate the recorded diet (including the timing of eating), and abstain from alcohol consumption 24 hours prior to each intervention. The intervention session will commence only if participants can verify their compliance with these protocols.

***Intervention sessions***

Figure 1 summarizes the timetable of events during each of the intervention sessions. The following timetable of measurements will occur during every intervention session: Participants will arrive in the clinic at about 7:30 am (when more than one participant is being tested on the same day, arrival times will be staggered to allow time for measurements to be completed on each participant), following a 10 h fast (including abstinence from all caffeinated beverages). The participant will then be asked to rest quietly for 10 min. Once the participant has been supine comfortably for at least 10 min a baseline measure of popliteal artery (PopA) hemodynamics, flow-mediated dilation (FMD) and blood pressure will be conducted. Immediately following these measurements, a cannula will be inserted into a forearm vein to allow for collection of multiple blood samples. The participant will then rest comfortably for at least 15 min before a baseline, fasting blood sample is collected. The participant will then be fed a standardized breakfast (0 min), with a snack provided at 240 min. Superficial popliteal artery haemodynamics and blood pressure will be measured, and blood samples collected hourly, with additional blood samples collected at 30 and 45 min after the consumption of the meal and snack. Flow mediated dilation will be measured with the participant in a supine position at baseline and completion of each intervention. Bathroom breaks will be scheduled to occur directly after haemodynamic measurements and blood collection at 90 min and 315 min – if the participant does not require use of the bathroom at these times they will still be encouraged to walk to the bathroom and back to ensure the amount of movement is the same between interventions.

Regular activity breaks intervention: At 30 min participants will walk on the treadmill at 5 km/h (~ average walking speed) for 2 min. They will then walk at the same speed for 2 min every 30 min until the intervention is completed (a total of 24 min of walking). Participants will remain seated between each walk on the treadmill.

Continuous Sitting: Participants will remain seated throughout the day, only moving from their chair for the scheduled bathroom breaks

Continuous standing: Beginning at 30 min participants will stand at a standing workstation continuously. All measurements will also be conducted in a standing position except the measurements conducted at 360 min.

Upon completion of the intervention session participants will be free to leave the laboratory, but will continue to wear both the accelerometer and the glucose monitoring system for 48 h after the intervention. At completion of the 48 h period participants will return to the clinic to have accelerometer and glucose monitor removed.


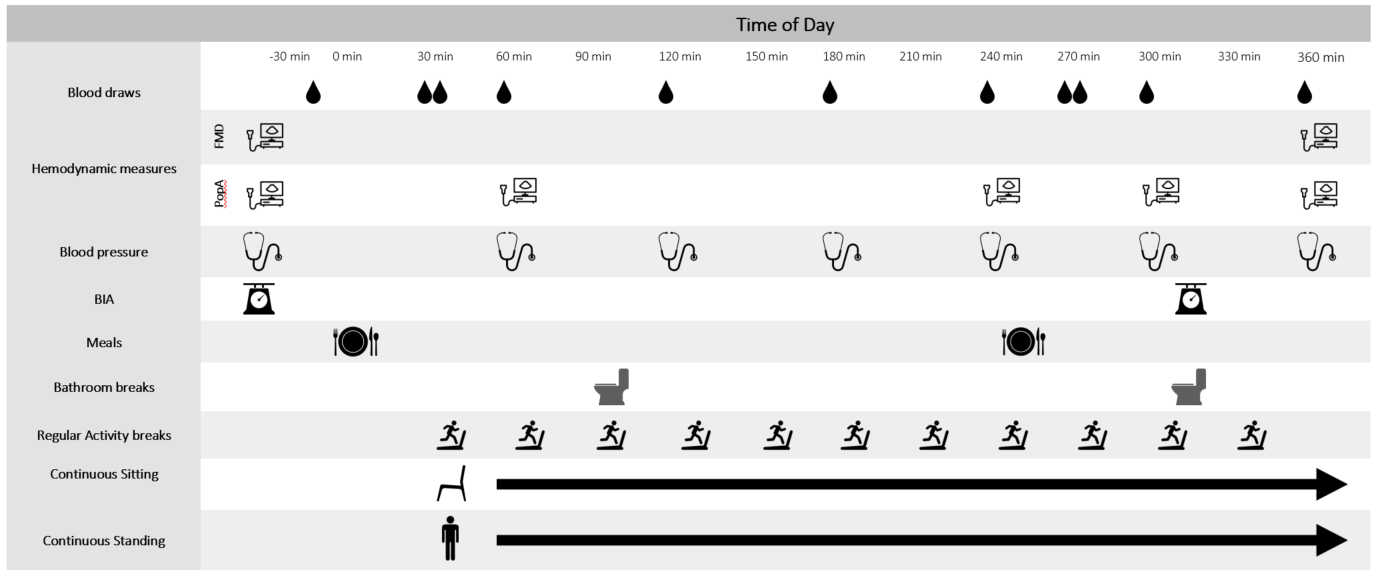


**Figure One:** Intervention session timelines

Measurements

***Measurement of superficial popliteal artery haemodynamics***

Popliteal artery (PoPA) diameter and blood velocity will be measured using ultrasound (Terason uSmart 3300, MA, USA) with a 15 MHz lineal array transducer (bandwidth 4-15 MHz) by simultaneously recording a longitudinal section B-mode image and a spectral Doppler trace of blood velocity. The Doppler angle of insonation will be maintained at ≤60°. Measurements will be made in the popliteal fossa with the participant in a semi-recumbent position, in the prolonged sitting and regular activity breaks interventions, and in a standing position in the prolonged standing intervention. The position will be marked on the skin with permanent ink to ensure the same location is used for each measurement. Locations will be measured from bony landmarks and/or the knee skin crease and replicated between protocols. Ultrasound depth, focus position and gain will be optimised for each participant for the first measurement and the same settings used each subsequent measurement. Video clips will be recorded using Camtasia Studio Screen Recording Software (TechSmith, MI, USA). Analysis of diameter and velocity, and calculation of shear rate will be performed using wall-tracking software (Cardiovascular Suite v 3.4, Quipu, Pisa, Italy). Haemodynamics will be assessed for ~60 seconds on each occasion. Ultrasound scans will be performed by one of the investigators (KT), and possibly one of the research students who will have extensive training in vascular sonography – however all the measurements performed on a participant (across all interventions) will performed by the same sonographer.

***Measurement of Flow-Mediated Dilation (FMD*)**

Flow-mediated dilation is a measure of endothelial function, which is based on the ability of the vessel to vasodilate in response to transient ischaemia. Transient ischaemia will be induced by inflating a 12 cm cuff around the mid-calf 200 mm Hg within 2 seconds (SC12 contoured leg cuff, E20 Rapid Cuff Inflator and AG101 Cuff Inflator Air Source, Hokanson, Bellevue WA, USA). Occlusion will be maintained for 5 min and recording will be continued for 3 min following rapid release of the cuff. Baseline diameter (D_base_), blood flow velocity (v) and shear rate (SR) will be calculated as the mean of the last minute of recording prior to cuff inflation. Peak diameter post-deflation will be determined automatically using the edge-detection software. Flow-mediated dilation will be calculated as the percentage increase in diameter from the baseline (FMD = (D_peak_-D_base_) / D_base_ x 100), following guidelines utilising allometric scaling to adjust for D_base_ with a covariate-controlled approach (24)

***Analytical methods***

Haematocrit will be measured from whole blood to estimate changes in blood volume as a result of each intervention session. The rest of the blood sample will be centrifuged within one hour of collection, and plasma stored at -80°C until analysis. Insulin analysis will be carried out using the Electrochemiluminescent Immunoassay (1010 Immunoassay System, Roche Diagnostic Elecsys^®^, Mannheim, Germany). Blood glucose concentration will be determined using enzymatic colorimetric methods (Roche diagnostics). Both incremental and total area under the curve will be calculated for plasma glucose, and insulin, using the trapezoidal rule, and used as summary measures.

Data Monitoring and Quality Control

No formal data monitoring will take place.

Quality control will be ensured by developing standard operating procedures for all data collection, including:

- The consent and screening visit (Anthropometry and blood pressure measurements, administration of health and demographic questionnaire)
- All measurements conducted during each of the three intervention sessions (meal preparation and timing of administration, timing of blood collection, timing of activity breaks)
- All vascular sonography, including measurements performed from recorded images
- Biological specimen management and analysis (tube labeling, centrifuging, separating of serum for storage in freezer, analysis of glucose and insulin concentrations)

All student researchers involved in the research will be thoroughly trained in all operating procedures and the PI (MP) will perform periodic checks throughout data collection to ensure protocols are being adhered to.

**12. Statistical Considerations and Data Analysis**

Sample size and statistical power

A sample size of 18 participants will provide 80% power to detect a 15% difference in D_base_-adjusted FMD (α = 0.05), and allow even numbers of participants to complete the three intervention in the six possible intervention orders. This sample size will also allow 80% power to detect a 10% difference in glucose AUC and a 15% difference in insulin AUC.

Statistical methods

Statistical analysis will be performed using STATA version 15 for Mac. Both incremental and total area under the curve will be calculated for plasma glucose and insulin, using the trapezoid rule, and used as summary measures. Mixed model regression will be used to examine between intervention changes in FMD and D_base_-adjusted FMD and glucose and insulin AUC controlling for any possible period or order effects.

**13. Ethical Considerations**

The study will be conducted in full conformance with the most current revision of the Declaration of Helsinki, the International Conference for Harmonization of Good Clinical Practice Regulations and Guidelines and the laws and regulations of New Zealand.

Quality assurance

This research will be conducted by researchers who are skilled in the technical aspects of this research study. Students working on the research project will be carefully trained and overseen by the named invesitgiators. Standard operating procedures will be developed and adhered to at all times.

Risks/safety considerations

***Cannula insertion and blood collection***.

There is a risk of discomfort, pain and bruising from the cannula insertion. Participants will be informed of the risks, and an experienced, nurse or phlebotomist will insert the cannula, and ensure strict aseptic technique is followed during blood collection from the cannula to minimize any risk of infection.

While most participants tolerate blood collection from a cannula very well, there are a small percentage of participants who may feel faint before and after collection is occurring, particularly during the continuous standing intervention. A research assistant will stand behind participants during blood collection, and if necessary assist them to sit if they feel light headed during collection. A chair will be within arm’s reach of the participant at all times while standing in case the feel faint at any time.

***Measurement of Flow-medicated dilation***

There is a risk of discomfort and pain during measurement of vascular function due to the cuff compression of the calf. This measurement will only be conducted if the procedure (and associated discomfort and pain) is tolerable to the participant.

***Treadmill walking***

There is a small risk of tripping when walking on the treadmill. Investigators will check participant’s shoe laces are tied prior to every walk on the treadmill, and will stand beside the participant when they are walking, and push the emergency stop button if required

Participants will be free to withdraw from the study at any time without any disadvantage to them.

Potential Benefits

Very little is understood about how using regular activity breaks and/or standing to interrupt prolonged sitting may impact endothelial function, despite the critical importance of endothelial function for acute and chronic perfusion on downstream tissues. This study will provide high quality experimental evidence around the effects of interrupting sedentary behaviour with short bouts of light intensity activity, or standing on endothelial function and glucose metabolism. Informing the development of future public health and physical activity guidelines.

Informed Consent

Participants who have indicated that they meet the eligibility criteria for the study via email will be asked to attend a consent and screening visit. Upon arrival the postgraduate research students or the PI (MP) will explain the study to the potential participant face to face. Participants will be asked if they have read the information sheet, and if they haven’t will be given the opportunity to read it. Potential participants will then be asked if they have any questions about participating in the study, which will be answered by the researcher conducting the consent and screening visit.

Written informed consent will be collected from the participant before the begin any further screening procedures.

*Completed consent forms will stored in the PIs (MCP) office in locked filing cabinet***.**

Participant Confidentiality

Upon enrolment, the participant will be assigned a unique identifying code consisting of “SSB” at the beginning, followed by 2 numbers (e.g. SSB01). To preserve confidentiality, during data-collection any paper-copy questionnaires will be identified only by the ID number. Any information linking the participant’s identity to the ID number will be kept in a password protected computer file.

ID numbers will be written on all survey documentation:

- The consent form
- The “PARTICIPANT ID:” space of the questionnaires
- Recording sheets from intervention sessions
- All biological sample tubes

Participant Follow-up

Once the data from the study have been analyzed, the participants will be provided with an overall summary of the results. Participants are also free to request a copy of their individual data.

Any participant who is identified as having high blood pressure or high fasting glucose as a result of the measurements performed during the study will be provided a written copy of these results, and advised to see their general practitioner to speak to them about their results.

Data Management

Data will be collected as per Standard Operating Procedures, and cleaned as per standard data entry procedures. Data quality checks will be run on all entered data to check for accuracy, consistency and completeness.

In the database the contains the results of the study each participant will be represented by an ID number. This database will be stored on the investigators computers, all of which are password protected. A backup copy may also be stored on the University’s shared server space, but only the PI (MCP) will have the password that will enable access to the data stored on the sever.

The file linking participants to their ID number will be stored in a separate password protected file that only the PI (MCP) will have access to. The only reason this information will be accessed once the study in completed is if the participant requests their individual results. This file will be destroyed once all participants have been given the opportunity to request individual information. The deidentified information collected as part of this research will be retained for at least 10 years in secure storage.

**14. Outcomes and Significance**

Completion of study proposed here will provide important high-quality evidence around how reducing sedentary behaviour by standing, or performing short bouts of regularly performed activity may facilitate improvements in both postprandial metabolism and endothelial function, both of which are established risk factors for cardio-metabolic disease. Given that the USA 2018 Physical Activity Guidelines Advisory Committee have recently highlighted the lack of high-quality evidence currently available around the interactions between sedentary behaviour and light and moderate intensity physical activity on health, this research clearly fills a gap in the literature that is of high importance to policy makers. It is anticipated that the outcomes of this research will help to inform public health messages and physical activity guidelines both in New Zealand and internationally.

**15. References**

1. Sedentary Behaviour Research Network. Letter to the editor: standardized use of the terms "sedentary" and "sedentary behaviours". Applied Physiology Nutrition and Metabolism 2012;37(3):540-2.

2. Keown M, Skeaff M, Perry T, Haszard J, Peddie M. Device Measured Sedentary Bheaviour Patterns in Office Employees. Journal of Occupational and Environmental Medicine 2018;In press.

3. Dunstan D, Barr E, Healy G, Salmon J, Shaw J, Balkau B, Magliano D, Cameron A, Zimmet P, Owen N. Television Viewing Time and Mortality: The Australian Diabetes, Obesity and Lifestyle Study (AusDiab). Circulation 2010;121:384-91.

4. Grøntved A, Hu F. Television Viewing and Risk of Type 2 Diabetes, Cardiovascular Disease, and All-Cause Mortality: A Meta-Analysis. Journal of American Medical Association 2011;305:2448-55.

5. Ekelund U, Steene-Johannessen J, Brown WJ, Fagerland MW, Owen N, Powell KE, Lee I-M, Lancet Physical Activity Series 2 Executive Committee, Lancet Sedentary Behaviour Working Group. Does physical activity attenuate, or even eliminate, the detrimental association of sitting time with mortality? A harmonised meta-analysis of data from more than 1 million men and women. The Lancet 2016;388(100051):1302-10.

6. Peddie M, Bone J, Rehrer N, Skeaff C, Gray A, Perry T. Breaking prolonged sitting reduces postprandial glycemia in healthy, normal-weight adults: a randomized crossover trial. American Journal of Clinical Nutrition 2013;98(2):358-66. doi: 10.3945/ajcn.112.051763.

7. Dempsey PC, Larsen RN, Sethi P, Sacre JW, Straznicky NE, Cohen ND, Cerin E, Lambert GW, Owen N, Kingwell BA, et al. Benefits for Type 2 Diabetes of Interrupting Prolonged Sitting With Brief Bouts of Light Walking or Simple Resistance Activities. Diabetes Care 2016;39(6):964-72.

8. Dunstan D, Kingwell B, Larsen R, Healy G, Cerin E, Hamilton M, Shaw J, Bertovic D, Zimmet P, Salmon J, et al. Breaking Up Prolonged Sitting Reduces Postprandial Glucose and Insulin Responses. Diabetes Care 2012;35:976-83.

9. Larsen R, Kingwell B, Robinson C, Hammond L, Cerin E, Shaw J, Healy G, Hamilton M, Owen N, Dunstan D. Breaking up of prolonged sitting over three days sustains, but does not enhance, lowering of postprandial plasma glucose and insulin in overweight and obese adults. Clinical Science 2015;129(2):117-27.

10. Chau JY, McGill B, Freeman B, Bonfiglioli C, Bauman A. Overselling Sit-Stand Desks: News Coverage of Workplace Sitting Guidelines. Health Commun 2017:1-7.

11. Saunders TJ, Atkinson H, Burr J, MacEwen B, Skeaff M, Peddie M. The acute metabolic and vascular impact of interrupting prolonged sitting: a systematic review and meta-analysis. Sports Medicine 2018;48(10):2347-66.

12. Thorp AA, Kingwell B, Sethi P, Hammond L, Owen N, Dunstan DW. Alternating Bouts of Sitting and Standing Attenuates Postprandial Glucose Responses. Medicine & Science in Sports & Exercise 2014:1. doi: 10.1249/MSS.0000000000000337.

13. Buckley JP, Hedge A, Yates T, Copeland R, Loosemore M, Hamer M, Bradley G, Dunstan DW. The sedentary office: a growing case for change towards better health and productivity. Expert statement commissioned by Public Health England and the Active Working Community Interest Company. British Journal of Sports Medicine 2015:1-7.

14. Dempsey PC, Blankenship JM, Larsen RN, Sacre JW, Sethi P, Straznicky NE, Cohen ND, Cerin E, Lambert GW, Owen N, et al. Interrupting prolonged sitting in type 2 diabetes: nocturnal persistence of improved glycaemic control. Diabetologia 2017;60(3):499-507.

15. Paing AC, McMillan KA, Kirk AF, Collier A, Hewitt A, Chastin SFM. Dose-response between frequency of interruption of sedentary time and fasting glucose, the dawn phenomenon and night-time glucose in Type 2 diabetes. Diabetic Medicine 2018;28:412-7. doi: 10.1111/dme.13829.

16. Green D. Exercise Training as Vascular Medicine: Direct IMporats on the Vasculature in Humans. Exercise and sport sciences reviews 2009;37(4):196-202.

17. Davignon J, Ganz P. Role of endothelial dysfunction in atherosclerosis. Circulation 2004;109(23 Suppl 1):III27-32.

18. Thosar SS, Johnson BD, Johnston JD, Wallace JP. Sitting and endothelial dysfunction: The role of shear stress. Medical Science Monitor 2012;18(12):RA173-80.

19. Ahmadi Abhari S, Sabia S, Shipley MJ, Kivimäki M, Singh Manoux A, Tabak A, McEniery C, Wilkinson IB, Brunner EJ. Physical Activity, Sedentary Behavior, and Long-Term Changes in Aortic Stiffness: The Whitehall II Study. Journal of the American Heart Association 2017;6(8).

20. Thosar SS, Bielko SL, Mather KJ, Johnston JD, Wallace JP. Effect of prolonged sitting and breaks in sitting time on endothelial function. Medicine and Science in Sports and Exercise 2015;47(4):843-9.

21. Morishima T, Restaino RM, Walsh LK, Kanaley JA, Padilla J. Prior exercise and standing as strategies to circumvent sitting-induced leg endothelial dysfunction. Clinical Science 2017;131(11):1045-53.

22. Krause N, Lynch JW, Kaplan GA, Cohen R, Salonen R, Salonen J. Standing at work and progression of carotid atherosclerosis. Scandinavian Journal of Enviromental Health;26(3):227-36.

23. Dempsey PC, Owen N, Yates TE, Kingwell B, Dunstan DW. Sitting Less and Moving More: Improved Glycaemic Control for Type 2 Diabetes Prevention and Management. Current Diabetes Reports 2016;16(114).

24. Atkinson G, Batterham A. The percentage flow-mediated dilation index: a large-sample investigation of its approriateness, potiential for bias and causal nexus in vascular medicine. Vascular Medicine 2013;18(6):354-65.
