# Supplementary material for: The effects of prolonged sitting, prolonged standing, and activity breaks on vascular function, and postprandial glucose and insulin responses: A randomised crossover trial
Source: PLoS One. 2021 Jan 4;16(1):e0244841. doi: 10.1371/journal.pone.0244841 (PMC7781669; doi:10.1371/journal.pone.0244841)
Supplement: Attachment — Submitted filename: Rev_Pone-D-20-21865.docx [file pone.0244841.s004.docx]

MS: PONE-D-20-21865
The study presented by Meredith C Peddie and coll. seems to be of interest; indeed, this question raised on the sedentary behavior, but also on the work-related constraints, could highlight a wider public health problem.

The paper is overall well written, and the study protocol seems to be well conducted.

Some question about:

Is your standard study protocol brand new or is it a protocol already used or tested, e.g. to evaluate other aspects in other studies?

About Meals: were food and drinks standardized anyway, or enrolled subjects were free to drink and eat anything? The author listed what the participants consumed, but it is unclear if each participant consumed all they listed consistently.

About Flow Mediated Dilation (FMD): authors didn’t specified the site of measurement.

About statistics: the statistical approach was not specified. Please consider the variable(s) distribution and sample size(s).

“Meals were fed at 0 and 240 min”: can the authors derive any implication –mainly on the insulin incretion – of 4-hrs fasting? Any to be corrected for in the statistics in order to evaluate the effect size of the intervention?

“This is the first study to measure the time course of the vascular and metabolic responses to Prolonged Sitting, Prolonged Standing and Regular Activity Breaks, and after consumption of a high-carbohydrate meal”. As general concept, since the authors did not mention the pulse wave velocity nowhere in the text, I think the this aspect deserve at least to be introduced.

“or the two toilet breaks were enough to mitigate the effects of sitting for long periods”: can you specify whether ALL participants had two allowed toilet breaks?

Last: I think the very strength of this study is to translate the protocol in suggestions for healthy and unhealthy people, but also for workers and employers.

Please thoroughly check your manuscript to fix any typo and grammar error.
